# Does the Quality of the Working Alliance Predict Treatment Outcome in Online Psychotherapy for Traumatized Patients?

**DOI:** 10.2196/jmir.8.4.e31

**Published:** 2006-12-19

**Authors:** Christine Knaevelsrud, Andreas Maercker

**Affiliations:** ^2^Institute of PsychologyUniversity of ZurichZurichSwitzerland; ^1^Treatment Centre for Torture VictimsBerlinGermany

**Keywords:** Online therapy, Internet, working alliance, therapeutic relationship, psychotherapy, psychotherapeutic processes, professional-patient relations, treatment outcome

## Abstract

**Background:**

The provision of online counseling and online therapy is steadily increasing. The results of a number of controlled trials investigating the efficacy of online approaches indicate that some of these new treatment alternatives might indeed be effective. Yet, little is known about how the therapeutic relationship (or working alliance) evolves over the Internet and whether it influences treatment outcome as it does in traditional face-to-face therapy.  The working alliance has been defined as the extent to which a patient and a therapist work collaboratively and purposefully and connect emotionally.

**Objective:**

The aim of the study was to investigate the quality and predictive relevance of the therapeutic alliance for patients receiving a short-term, Internet-based, cognitive-behavioral therapy program for posttraumatic stress reactions.

**Methods:**

After rigorous screening for exclusion criteria of high dissociative tendencies, risk of psychosis, and suicidal tendencies, 48 patients, who had experienced a traumatic event in the past, were included in the online treatment study. The short form of the Working Alliance Inventory (WAI-S) was administered at the fourth treatment session. The relevance of the therapeutic relationship for treatment outcome was assessed in terms of residual gain from pretreatment assessment to the end of treatment. The revised Impact of Event Scale (IES-R) and the depression and anxiety subscales of the Brief Symptom Inventory (BSI) were used to assess treatment outcome.

**Results:**

A total of 48 participants were included in the analysis. Overall, high alliance scores were found. In contrast to previous studies of conventional face-to-face therapy, there was only a low to modest association (.13 to .33) between the quality of the therapeutic relationship and treatment outcome.

**Conclusion:**

High alliance scores indicate that it was possible to establish a stable and positive therapeutic relationship online. However, the therapeutic relationship was found to be a less relevant predictor of the therapy outcome than in face-to-face approaches. We discuss whether this finding can be attributed to methodological reasons such as the restricted range of alliance ratings obtained or the time of administration of the WAI-S, or whether the therapeutic relationship might be less relevant to the treatment outcome of online therapy approaches.

## Introduction

Recent developments in communication technology have opened up new therapeutic possibilities that challenge our understanding of psychotherapy. While the academic debate continues as to whether online treatments might present an acceptable alternative to face-to-face therapy, real life has already decided. “Researchers can no longer discuss online counseling as an intervention method that will take shape in the future—the future is now” [1]. Internet-based treatment approaches have already been developed for a wide range of clinical disorders including depression, eating disorders, anxiety disorders, and substance abuse, as have interventions targeting relationship problems, adjustment disorders, and work-related burnout, and the numbers are expected to increase [2]. The numbers of empirical studies investigating the efficacy of online approaches are growing apace, and results indicate that some of these new treatment alternatives might indeed be effective (see [3] for a review). However, an important question remains largely unanswered: What contributes to therapeutic change? To date, virtually no studies have focused on the processes underlying online therapy [4]. Thus, it is not clear whether online therapy is based on factors and mechanisms similar to those that are responsible for therapeutic change in face-to-face therapy or whether we need to redefine our understanding of the underlying processes when considering online therapy.

### The Therapeutic Relationship

The quality of the therapeutic relationship, or working alliance, has been demonstrated to be especially important in predicting the outcome of psychotherapy. The working alliance has been defined as the extent to which a patient and a therapist work collaboratively and purposefully and connect emotionally [5]. Research reviews have consistently reported a positive relationship across studies between the quality of the therapeutic alliance and therapy outcome, although there are some instances where the working alliance fails to predict outcome or where associations are nonsignificant [5-10]. In their meta-analysis, Martin et al [10] reported that the quality of the therapeutic alliance accounted for 22% of the variance in the rate of therapeutic success. Moreover, research has indicated that the relationship between therapeutic alliance and treatment outcome holds across several types of treatment, including cognitive-behavioral therapy (CBT) [11], interpersonal therapy [9], and psychodynamic therapy [7,11], and does not differ significantly within treatment approaches [8,9].

### Online Relationships

The beneficial effects and clinical relevance of a positive working alliance have been well documented in face-to-face therapies, but almost nothing is known about how the therapeutic relationship operates online. Online therapy challenges our basic assumptions about what is needed to establish a therapeutic contact, such as (1) sharing the same physical space, (2) talking, and (3) synchronous real-time interaction [12], and it is still uncertain if online therapy provides conditions that are sufficient to establish a stable therapeutic alliance at all. Since one of the major criticisms of online therapy concerns the ambiguous nature of the therapeutic relationship, research in this field is needed. Most previous studies have focused on relational behavior in everyday online contact, with inconsistent results. These findings prompted an academic discussion between proponents of two contrasting views of the online relationship. On the one hand, Slouka [13] states that online relationships are shallow, impersonal, and unreal. Indeed, Kraut et al [14] have demonstrated that online relationships heighten depression and loneliness rather than provide fulfilling relationships. Mallen et al [1] compared Internet-based and face-to-face conversations in a randomized study and found that participants who communicated online felt less satisfied with their contact and experienced a lower degree of self-disclosure and closeness with their partner than participants in the face-to-face group. On the other hand, various other authors have shown that online contacts are just as real and intense as face-to-face relationships and that differences between online relationships and face-to-face relationships diminish over time [15]. Whitty and Gavin [16] found that the absence of social clues enhanced and encouraged the development of relationships. This is in line with prior research indicating that visual anonymity contributes to higher levels of self-disclosure and openness [17,18].

It should be noted, however, that online therapeutic contact differs markedly from arbitrary, anonymous online contact, the most important difference probably being the identity of the therapist. In online therapeutic contact, the address, telephone number, and credentials of both parties are accessible. Furthermore, the frequency of contact is predefined and there are set time limits for response. Thus, aspects such as uncertainty about the identity and honesty of the other party, which might be detrimental to establishing a trustful contact, are much reduced in online therapeutic relationships compared with anonymous online contacts.

Focusing on the working alliance online, Cook and Doyle [19] evaluated differences in client ratings of the working alliance between a small sample (N = 15) of online therapy clients and normative data from a comparable face-to-face counseling sample. They found comparable (and relatively high) evaluations of the working alliance in the online sample using the frequently applied Working Alliance Inventory (WAI) [20].

Lange et al [21] conducted an Internet-based treatment study of work-related burnout. After completing the course of treatment, patients where asked to rate the contact with their therapists: 75% of the 115 participants described the contact as personal and 88%, as pleasant; 80% rated being treated exclusively via the Internet as positive, and 70% indicated that they did not miss face-to-face contact. Cohen and Kerr [22] compared the impact of one session of face-to-face counseling with online counseling (chat) in terms of posttreatment anxiety and attitudes toward counseling. Participants (N = 24) were randomly assigned to one of the two experimental groups. Clients in both groups experienced a uniform decrease in anxiety and rated their counselors equally on expertness, attractiveness, and trustworthiness, regardless of the mode of delivery.

While data from the aforementioned studies provide valuable information and preliminary evidence that a positive working alliance can be developed through the Internet, empirical data derived from systematic exploration of the online therapeutic relationship remain sparse. Thus, it is essential to investigate whether it is possible to develop a therapeutic alliance in the absence of visual and auditory cues and whether the working alliance has the same predictive value in online treatment as in face-to-face therapy.

### Research Questions

The present study aims to replicate prior findings concerning the relationship between the working alliance and treatment outcome in face-to-face therapy. It was hypothesized that the baseline psychopathology would be inversely associated with the patients’ assessment of the therapeutic alliance. Furthermore, it was hypothesized that the quality of the online therapeutic alliance would predict the residual gain from pretreatment assessment to end of treatment. We expected the patients’ ratings of the alliance to be more highly correlated with therapy outcome than the therapists’ ratings, and the patients’ and therapists’ assessments of the therapeutic alliance to be only moderately related. Overall, we expected that it would be possible to establish a positive and stable therapeutic relationship online, characterized by high scores on the WAI. The present study is part of a larger study with random assignment to a treatment group or a waiting-list control group [Knaevelsrud and Maercker, in preparation]. Based on the research questions chosen, only the data from the treatment group were used in the following analyses.

## Methods

### Recruitment

Participants were recruited by means of radio and newspaper advertisements as well as advertisements posted on websites for different groups (eg, crime victims, sexual abuse victims, bereaved parents). To be included in the study, participants had to (1) have experienced a traumatic event that occurred at least one month prior to treatment and that met the criteria specified in the DSM-IV [24], (2) be 18 years or older, (3) not exceed the cutoff scores for dissociation and psychosis (see exclusion criteria), (4) not abuse alcohol or other drugs, (5) not consume neuroleptics, (6) be fluent in written German, and (7) not be receiving treatment elsewhere.

A total of 498 potential participants showed interest in the treatment; 68% (N = 337) returned the screening questionnaires; 73% (N = 246) were excluded based on the exclusion criteria. In total, 91 patients participated in this study (48 in treatment group; 43 in control group).

Potential patients browsed through the Interapy website, which provided information about (1) posttraumatic stress reactions, (2) the study and its inclusion criteria, (3) the treatment, (4) the therapists and supervisors, and (5) other treatment alternatives. Potential participants were sent screening questionnaires by email. Those who passed the screening received an informed consent document. Participants were required to sign and return this document, indicating that they had been informed about the aim and procedures of the research project and were willing to take part in it. Based on a computer-generated randomization list, they were randomly assigned to the waitlist-control group or treatment group. Patients who were excluded from the study were provided with information on where they could receive treatment elsewhere.

To gather miscellaneous information, including the time since the trauma, education level, degree of computer and Internet experience, and typing skills, a short checklist was administered.

### Therapists

Two therapists conducted the treatment. Both were female psychologists who had received special training in the application of writing assignments for the treatment of posttraumatic stress disorder (PTSD). One was trained in cognitive-behavioral psychotherapy. Their average age was 33 years. The therapists participated in weekly supervision sessions.

### Exclusion Criteria

#### Dissociation

Dissociative symptoms were tapped using the Somatoform Dissociation Questionnaire (SDQ-5) [25]. The scale consists of five items, which are rated on a 5-point Likert scale (1 = not at all, 5 = very often). The internal consistency of the SDQ-5 is good (α = .80). Participants who scored above the cutoff score were excluded from the treatment.

#### Risk of Psychosis

Risk of psychosis was measured using the Dutch Screening Device for Psychotic Disorder [26]. This seven-item inventory has high internal consistency (α = .82) and is a good predictor of psychotic episodes. In a Dutch study, a high level of agreement was found between the self-reports of 33 patients and their clinicians’ reports on them (α = .85). Since no German norm group exists as yet, the data from the Dutch norm group were used. Participants were excluded if they scored above the cutoff score. Participants were also excluded if they indicated the use of neuroleptics.

#### Risk of Suicide

Suicidal intentions and risk of suicide were measured using the Suicide Risk Assessment (SRA) [27], a six-item, self-report questionnaire designed to capture suicidal tendencies. It consists of questions tapping suicidal plans, previous suicide attempts, and current suicidal intentions.

#### Treatment

Patients were sent two weekly 45-minute writing assignments over a five-week period (10 essays in total). Before and after the treatment, participants completed a set of questionnaires measuring the treatment effect. The therapy consisted of three treatment phases: (1) self-confrontation, (2) cognitive reconstruction, and (3) social sharing. After the fourth writing session, which constituted the end of the first treatment phase, the short form of the WAI (WAI-S) was administered. The treatment procedure is described in detail by Lange et al [23] and will only be outlined in brief here.

#### First Phase: Self-Confrontation

At the beginning of the treatment, participants received psychoeducation on the mechanisms of exposure. In the first phase, the therapists helped the patients to focus on the most painful images and thoughts and encouraged the patients to write about them. The patients were instructed to describe the traumatic event thoroughly, including their intimate fears and thoughts concerning the traumatic experience. To increase the effect of the exposure, patients were asked to write in the first person and in present tense and to give detailed descriptions of all sensory details they experienced during the traumatic event, including olfactory, visual, and auditory stimuli. Participants were explicitly asked not to concentrate on style, grammar, spelling, or the chronological order of their essays. The therapists checked whether patients explicitly addressed the traumatic event as described above and, if needed, supported the patient to address the avoided features more forcefully.

#### Second Phase: Cognitive Restructuring

During the second phase, patients received psychoeducation on the principles of cognitive restructuring. The goal of this phase was to form a new perspective of the traumatic event and to regain a sense of control. Participants wrote a supportive letter to an imaginary friend who had been through the same experience. In this letter, the patient was instructed to reflect on the addressee’s feelings of guilt and shame, challenge dysfunctional automatic thinking and behavior patterns, and correct unrealistic assumptions. Furthermore, patients were encouraged to consider potentially positive consequences of the traumatic event for that person’s life and the lessons to be learned from it.

#### Third Phase: Social Sharing and Farewell Ritual

During the third phase, patients received psychoeducation on the positive effects of social sharing. In a final letter, they took symbolic leave of the traumatic event. Patients summarized what happened to them, reflected on the therapeutic process, and described how they were going to cope now and in the future. Patients could address the letter either to themselves, to a close friend, or another significant person involved in the traumatic event. The letter did not ultimately have to be sent.

**Figure 1:**
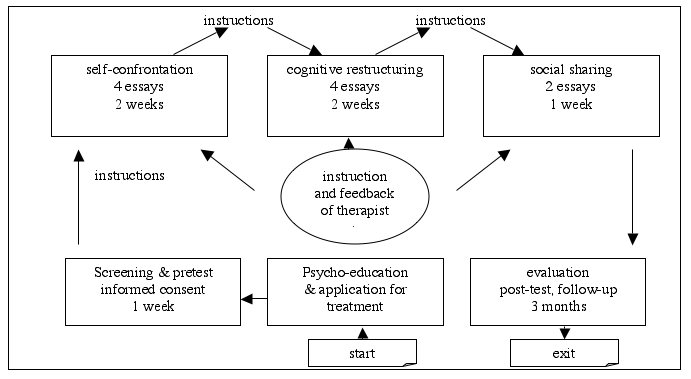
Overview of the Interapy procedure

At the beginning of each writing phase, patients proposed individual timetables as to when they planned to write. Halfway through and at the end of each treatment phase, patients received feedback and further writing instructions, which consisted of standard instructions and standard feedback tailored to patients’ specific needs. Important aspects of this feedback were recognition and reinforcement of the patients’ independent work, positive feedback, motivation and unconditional support, as well as frequent summaries and encouragement of patients to voice questions and doubts. An overview of the Interapy procedure is given in Figure 1.

### The Therapeutic Alliance

The WAI [20] is a transtheoretical measure of the working alliance that was designed to apply to diverse therapeutic orientations and modalities and is one of the questionnaires most frequently used to measure the working alliance [28]. In this study, the WAI-S [29] was used. Busseri and Tyler [28] have shown that the two versions correlate highly in terms of their psychometric and predictive qualities and are thus interchangeable. The WAI-S is a 12-item, self-report questionnaire consisting of three subscales designed to assess three primary components of the working alliance: (1) how closely client and therapist agree on and are mutually engaged in the goals of treatment (goal subscale reliability coefficient in this study:α = .79), (2) how closely client and therapist agree on how to reach the treatment goals (task subscale reliability coefficient in this study:α = .70), and (3) the degree of mutual trust, acceptance, and confidence between client and therapist (bond subscale reliability coefficient in this study:α = .75). The composite score (reliability coefficient in this study: = .83) is used as a global measurement of working alliance. Respondents were asked to rate each statement on a 7-point Likert scale ranging from 1 (never) to 7 (always).

### Outcome Measurements

To assess posttraumatic stress, the revised version of the Impact of Event Scale (IES-R) [30] was used. The scale consists of 22 items constituting the subscales of (1) intrusions, (2) avoidance, and (3) hyperarousal, the three main characteristics of psychological dysfunction after a traumatic life event. Participants were asked to indicate the frequency of each symptom over the past 7 days on a 4-point Likert scale. The presence of a PTSD diagnosis was assessed using the cut-off score proposed by Neal et al [31]. They found that an optimum cut-off score for the IES (which comprises the avoidance and intrusion subscales) of 35.0 produced the highest predictive value.

To measure depression and anxiety, the appropriate subscales of the short form of the Symptom Checklist (SCL-90), the Brief Symptom Inventory (BSI) [32], were used to measure the effects of treatment on psychological dysfunction in dimensions related to symptoms of posttraumatic stress. The two subscales consist of six items each. Each item is rated on a 5-point Likert scale (0 = not at all, 4 = extremely).

## Results

Statistical analyses were only performed on the data of the 48 participants in the treatment group. Participants in this group ranged from 18 to 68 years of age, with an average age of 35 years; 92% were female; 55% had a university degree, and a further 14% had a secondary school leaving certificate. The most frequently reported traumatic event was the sudden or violent death of a close person (40%); 38% of the patients reported sexual abuse, incest, or rape; 10% were crime victims. On average, the traumatic event had occurred 3.5 years previously (range 2-696 months).

Scores on the IES-R indicated that the 48 participants suffered greatly. The mean scores on the intrusions (mean = 23.1; SD = 6.5) and avoidance (mean = 19.4; SD = 9.9) subscales were in the upper regions of the norm table for Dutch PTSD patients [33].

### Dropouts

Those who terminated the treatment early (dropout 17%, N = 8) were compared with those who completed the program in terms of demographic variables. Chi-square analyses failed to reveal any significant differences between dropouts and completers in terms of gender, education level, or marital status, and a *t* test showed no significant differences in terms of age or years since the trauma. We also used *t* tests to assess differences between dropouts and completers in pretreatment psychopathology: no significant differences were found for depression (BSI) (*t*_46_ = .78, *P* = .44), anxiety (BSI) (*t*_46_ = .84, *P* = .41), posttraumatic symptoms (IES-R) (*t*_46_ = −1,077, *P* = .29), or WAI-S (*t*_41_ = −.639, *P* = .53). Note, however, that WAI-S scores were only available for three dropouts. The other dropouts terminated therapy before the WAI-S was administered.

### Patients’ Pretherapy Status and Ratings of Treatment Relationship

Focusing on the 40 patients who completed the course of therapy, zero-order Pearson correlations were used to assess the relations between variables (Table 1). Bivariate analysis of relationships between pretreatment psychopathology and working alliance scores revealed no significant pattern of relationships but showed a tendency of an inverse relationship.

**Table 1:** Means, standard deviations, and correlations of patients’ scores on the WAI-S (at 4th session^‡^) and initial symptoms (at 1st session^†^) (N = 40)

	**Mean**	**SD**	**Goal**^‡^	**Task**^‡^	**Bond**^‡^	**Composite**^‡^
**WAI-S**^‡^						
Goal	5.8	.77	1			
Task	5.7	.80	.90	1		
Bond	6.2	.75	.31	.15	1	
Composite	5.8	.62	.90	.83	.64	1
**IES-R**^†^						
Intrusions	24.4	6.2	.12	.04	−.11	.04
Avoidance	18.9	10.2	−.12	.11	−.35^*^	−.19
Hyperarousal	21.6	6.7	.09	.02	−.20	−.08
**BSI**^†^						
Anxiety	9.5	3.2	−.16	−.11	−.34^*^	−.26
Depression	10.4	4.0	.09	.01	−.13	−.04

^*^P < .05

^†^at 1^st^session

^‡^at 4^th^session

### Association of the Working Alliance With Therapy Outcome

As indices of client outcome, the residual gain scores on each subscale of the self-report measures (BSI, IES-R) were calculated. Each participant’s residual gain score at each posttreatment assessment point was the deviation of the posttreatment score on that measure from the pretreatment assessment. Residual gain scores were reversed as appropriate so that higher scores indicate greater improvement (ie, greater reduction in psychopathology). These residual gain scores across the patients were correlated with their scores on the WAI-S. Table 2 shows partial correlations between the patients’ scores on the WAI-S (subscales and composite) and their posttreatment scores on target variables (BSI, IES-R) after partialing out initial symptom levels.

**Table 2:** Means, standard deviations, and correlations of the WAI-S patient and therapist ratings and residual gain (N = 40)

			**WAI-S Patient Ratings**				**WAI-S Therapist Ratings**			
	**Mean**	**SD**	**Goal**	**Task**	**Bond**	**Composite**	**Goal**	**Task**	**Bond**	**Composite**
**IES-R residual gain**										
Intrusions	13.0	9.4	.15	.17	.01	.16	.08	.09	.05	.08
Avoidance	11.8	10.6	.22	.22	−.12	.13	.19	.25	−.22	.08
Hyperarousal	13.0	9.0	.09	.09	.13	.15	.03	−.08	−.05	−.04
**BSI residual gain**										
Anxiety	4.9	4.2	.27	.24	.19	.33^*^	.30	.27	.09	.25
Depression	6.0	4.4	.27	.29	−.03	.21	.17	.21	.13	.20

^*^P < .05

Positive correlations were found between the patients’ ratings of the working alliance and therapy outcome. However, with the exception of the relation between the WAI-S composite score and anxiety (*r* = .33, N = 40, *P* = .04), these correlations did not reach statistical significance. For the most part, positive correlations were also found between the therapists’ ratings of the working alliance and the outcome, although these did not reach statistical significance either. Multiple regression analyses were used to further explore possible mediator or suppressor effects of the working alliance on outcome variables (residual change in IES-R composite score and residual change in BSI anxiety and BSI depression). Results revealed that the working alliance, as rated by patients, did not exert a significant direct influence on posttraumatic symptoms (adjusted *R* ^2^ = −.026, F_1,38_ = .007, *P* = .93); depression (adjusted *R* ^2^ = −.026, F_1,38_ = .005, *P* = .94); or anxiety (adjusted *R* ^2^ = −.017, F_1,38_ = .358, *P* = .55).

## Discussion

The aim of this study was to investigate the quality and the possible influence of an Internet-based therapeutic relationship on treatment outcome. To our knowledge, this was the first study in which the effects of the working alliance have been systematically evaluated in an Internet-based therapy approach. Bearing in mind that the generalizability of our findings is limited by the small sample size and the narrow diagnostic range of clients, we now turn to the research questions raised above.

### Does Baseline Psychopathology Predict the Quality of the Working Alliance?

No significant relationship was detected between the severity of pretreatment psychopathology and the working alliance rating. However, a tendency of an overall inverse relationship was observed, indicating that patients who experienced more severe symptoms at the beginning of the treatment tended to have a less positive relationship with their therapist. This would be in line with previous research by Taft et al [34], who found a significant inverse correlation (*r* = −.31) between psychopathology and early working alliance ratings in face-to-face therapy.

### Is the Quality of the Therapeutic Alliance Linked to Treatment Outcome?

The results failed to confirm the hypothesis that a strong working alliance early in treatment would predict positive psychological changes later in treatment. However, almost all of the correlations were positive, indicating that residual gains on outcome measures were associated with higher rather than lower mean WAI-S scores, except in the relation between working alliance and anxiety. The finding that the WAI-S failed to predict therapy outcome in our sample stands in marked contrast to the findings for most face-to-face studies. This discrepancy may be attributable to a number of factors. One explanation for the lack of effect may be the almost uniformly high levels of alliance ratings (ie, restricted range) obtained in this study, perhaps due to the self-selected sample. Most of the patients were recruited through the Internet, which suggests that they were already comfortable with this medium. Research has shown that computer experience influences the way people judge Internet-based contact. In their study, Mallen et al [1] showed that the more familiar participants were with Internet-based contact, the more positively they judged that contact to be.

Another possible reason for the failure to find more substantial relationships between the quality of the working alliance and treatment outcome has been proposed by Stiles et al [11], who found great variability in the correlation with outcome measures taken at different stages in the therapy. They suggest that this might explain why various studies in which the working alliance was only measured on a single occasion produced inconsistent alliance/outcome correlations. This line of reasoning suggests that the question is not whether the working alliance is more important in a particular type of therapy, but rather whether the alliance is being measured in a way that is appropriate to that particular therapy. The time of administration of the WAI (in terms of the number of sessions) has been found to influence the rating of the working alliance [5,29]. It has also been suggested that treatment outcome may be particularly well predicted by the quality of the working alliance as measured in early sessions [5,8,11]. As is standard practice in face-to-face studies, the therapeutic alliance was assessed early in the therapeutic process in the present study, after the fourth writing session [28]. At that point, however, there had been only three therapist/client contacts, which may not in fact have been sufficient to evaluate the therapeutic alliance in online therapy. It could be that, given the different conditions under which the working alliance develops in Internet-based treatment approaches, administering the WAI later on in the therapy might yield more accurate measurements.

Alternatively, although the alliance has been shown to predict the outcome of other modes of delivery, it may not be a crucial factor in facilitating positive psychological change in Internet-based manualized therapies. The treatment applied in this study incorporates principles derived from CBT, with standardized instructions and a fixed treatment manual, and focuses on client empowerment and self-efficacy. It may be that the nonspecific factor of the therapeutic relationship played a less important role than it does in less structured face-to-face therapy.

### Can a Positive and Stable Relationship Be Established Through the Internet?

Patients reported high levels of therapeutic alliance early in treatment. The patients’ ratings of the therapeutic relationship in our study were even higher than in face-to-face studies. Hersoug et al [35] administered the WAI to 270 patients with multiple clinical disorders in the third session of a conventional face-to-face therapy approach. Compared to the mean composite score in their study (mean = 4.94; SD = 1.08), our patients’ ratings of the Internet-based relationship were more than one standard deviation higher (mean = 5.8; SD = 6.2), as shown in Table 1. The patients’ positive evaluation of the therapeutic relationship indicates that a therapeutic alliance can be established through the Internet. Furthermore, a strong working alliance can be expected to promote treatment adherence as assessed by factors such as dropout rates. Given that trauma victims have been shown to have compliance problems [36,37] and high dropout rates (up to 28%) [38], the high WAI-S ratings and the relatively low dropout rate (17%) in this study give reason to conclude that it was indeed possible to develop a positive and stable therapeutic relationship through the Internet. It must be noted, however, that the therapeutic contact as performed during the Interapy treatment is rather exceptional. The intensity of individualized support and regular personal interaction differs markedly from online approaches where online personalized communication is rather uncommon.

### Limitations

The following limitations necessitate caution in the interpretation of our results. First, the modest sample size may have provided insufficient power to uncover the complex interplay of the online working alliance and psychopathology measures.

Second, only 17% of applicants could be included in the study, which might limit the external validity of the present findings. The same applies to the specific sample of trauma victims. Trauma survivors have been noted by many clinicians to have difficulty in tolerating the interpersonal nature of therapy, particularly “the [need] to trust another person with his or her pain” [39] (p. 538). Given that trauma victims are especially prone to feelings of guilt and shame, they might be especially drawn to the medium of the Internet, where visual anonymity enables them to disclose painful and shameful details more easily than in face-to-face settings. Extending this research paradigm to clinical samples other than trauma victims could help to clarify this relationship.

Third, further research efforts should be initiated to address the possibility that the results are only valid for users who are already comfortable with the Internet due to self-selection. A direct comparison of an online intervention and a face-to-face intervention as a randomized controlled trial would be indicated to investigate how the text-based bond formed in online therapy compares and contrasts with the in-person therapeutic alliance.

Limitations notwithstanding, the findings presented here are of interest because they indicate that a stable and positive relationship can be established online, although the quality of the relationship does not predict treatment outcome. The rapid growth of Internet-based treatment approaches makes it likely that online therapies will become an enduring component of the psychotherapeutic landscape. One line of future research will be to identify predictors of a positive therapeutic relationship. A major challenge when building online relationships is to become aware of the nuances in the written language used in this context [12], which has accents, ambiguities, and individual styles, as well as the use of emoticons (emotion + icon; eg, a happy face ☺). Clinicians who work online should be given clinical training focusing on features of written communication. In addition, further work is needed to determine whether the role of the working alliance differs as a function of the mode of delivery, and to disentangle the relationships between the therapeutic alliance, specific cognitive-behavioral techniques, and treatment outcome. It also remains to be seen whether working alliance scores will predict long-term reductions in psychopathology rather than focusing on short-term changes in psychological functioning, as was the case in this study.

## Abbreviations

BSI: Brief Symptom Inventory
CBT: cognitive-behavioral therapy
DSM-IV: Diagnostic and Statistical Manual of Mental Disorders, Fourth Edition
IES-R: revised version of the Impact of Event Scale
PTSD: posttraumatic stress disorder
SCL-90: Symptom Checklist
SDQ-5: Somatoform Dissociation Questionnaire
SRA: Suicide Risk Assessment
WAI: Working Alliance Inventory
WAI-S: short version of the WAI
